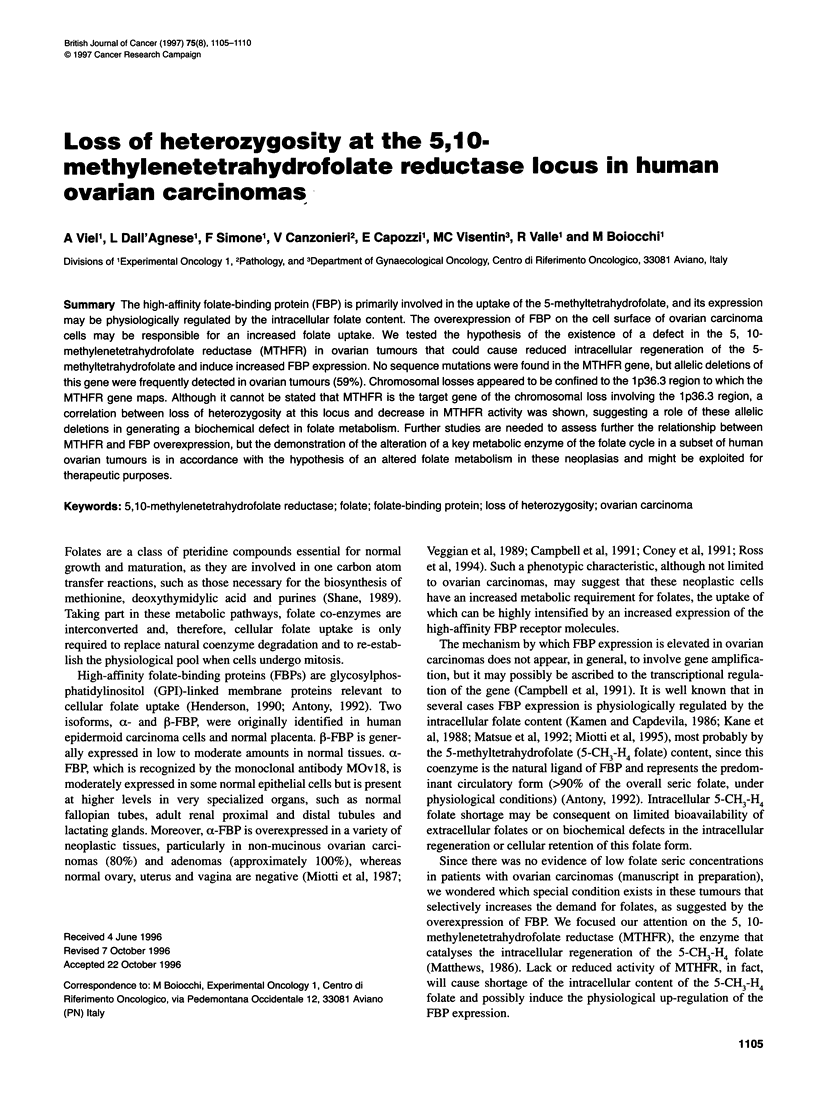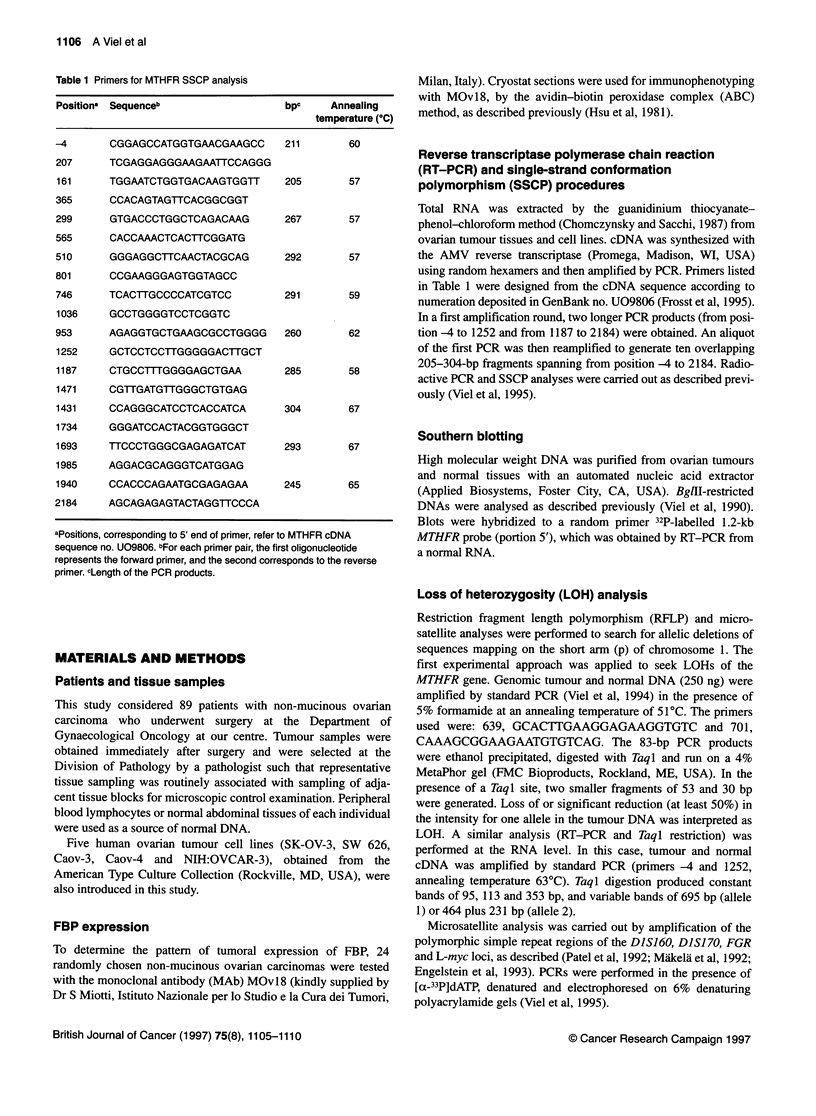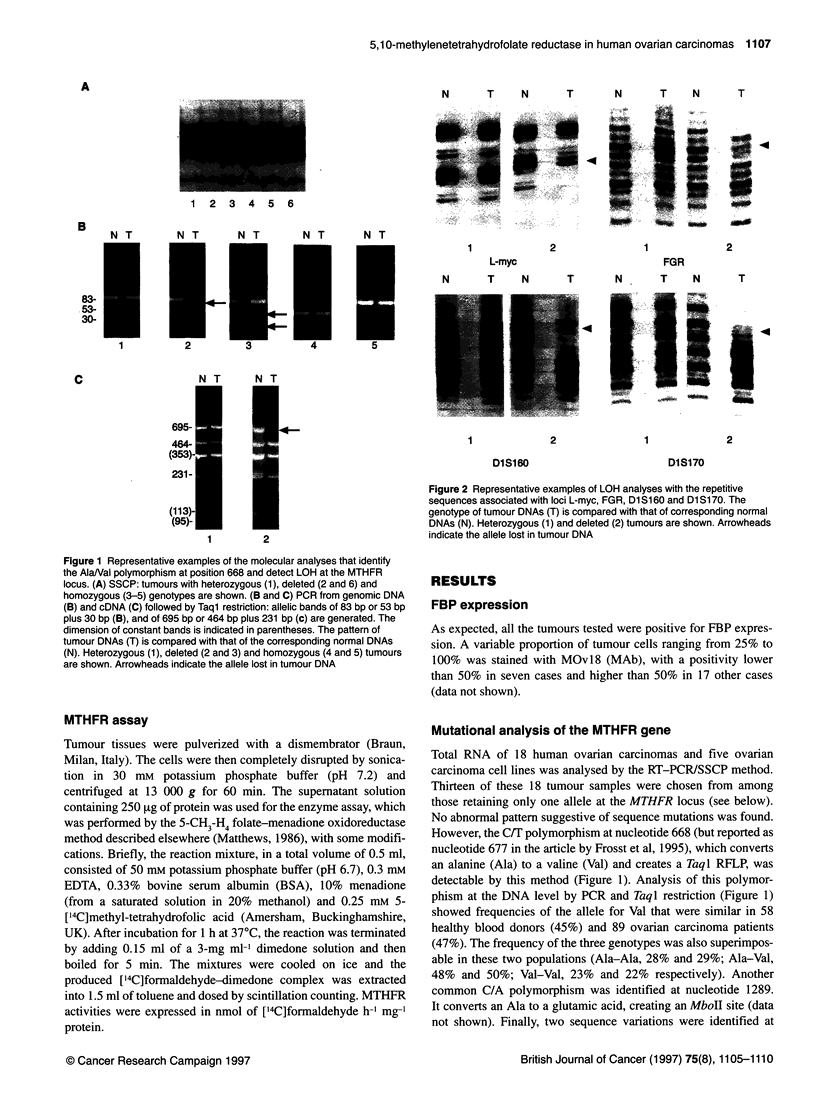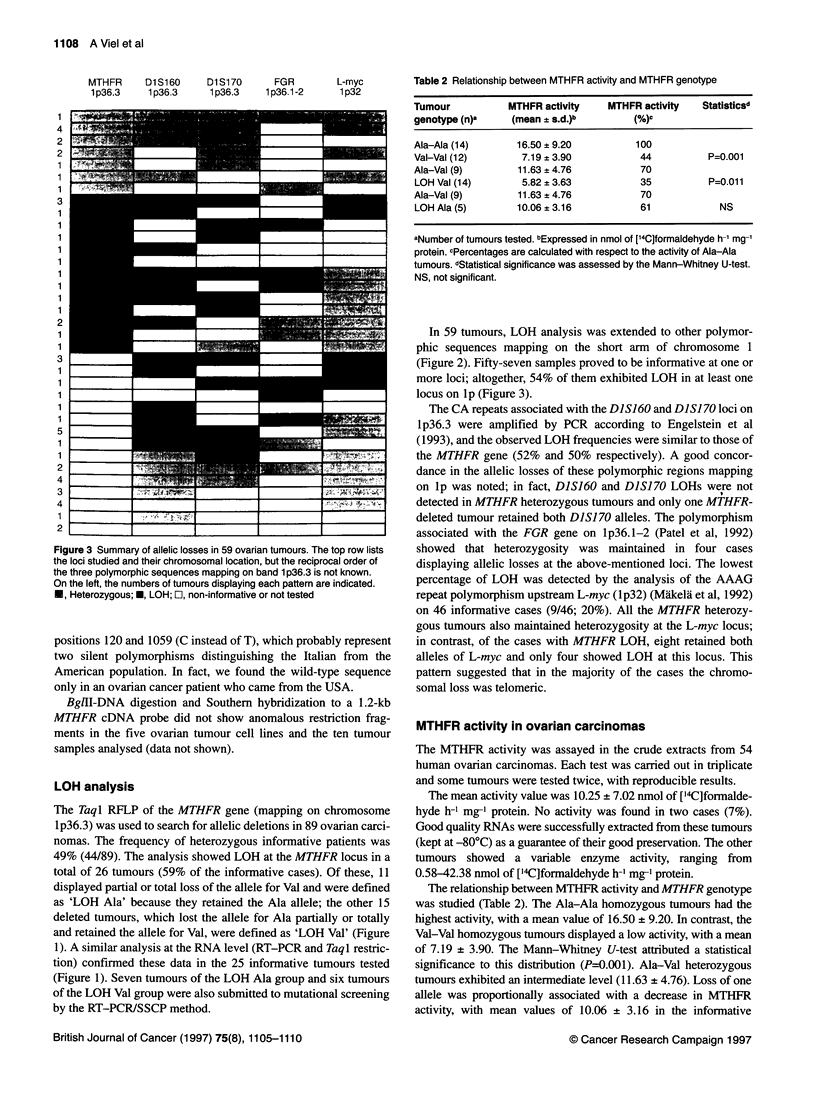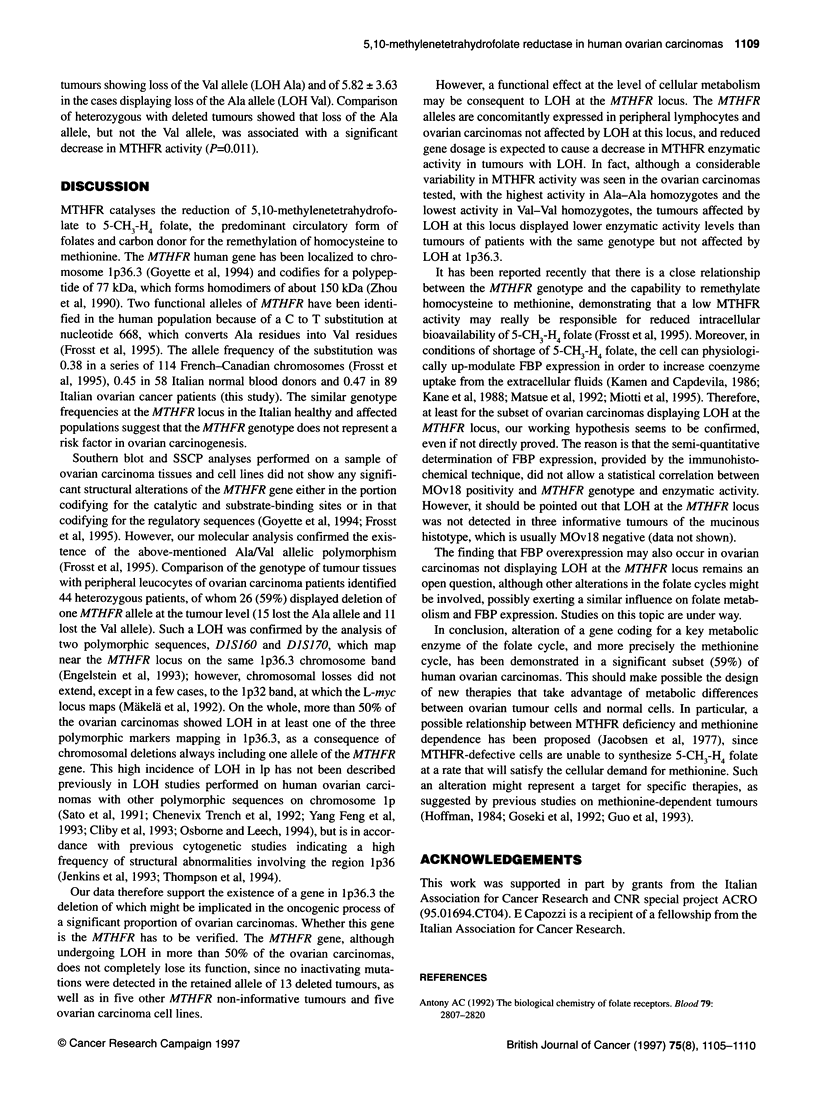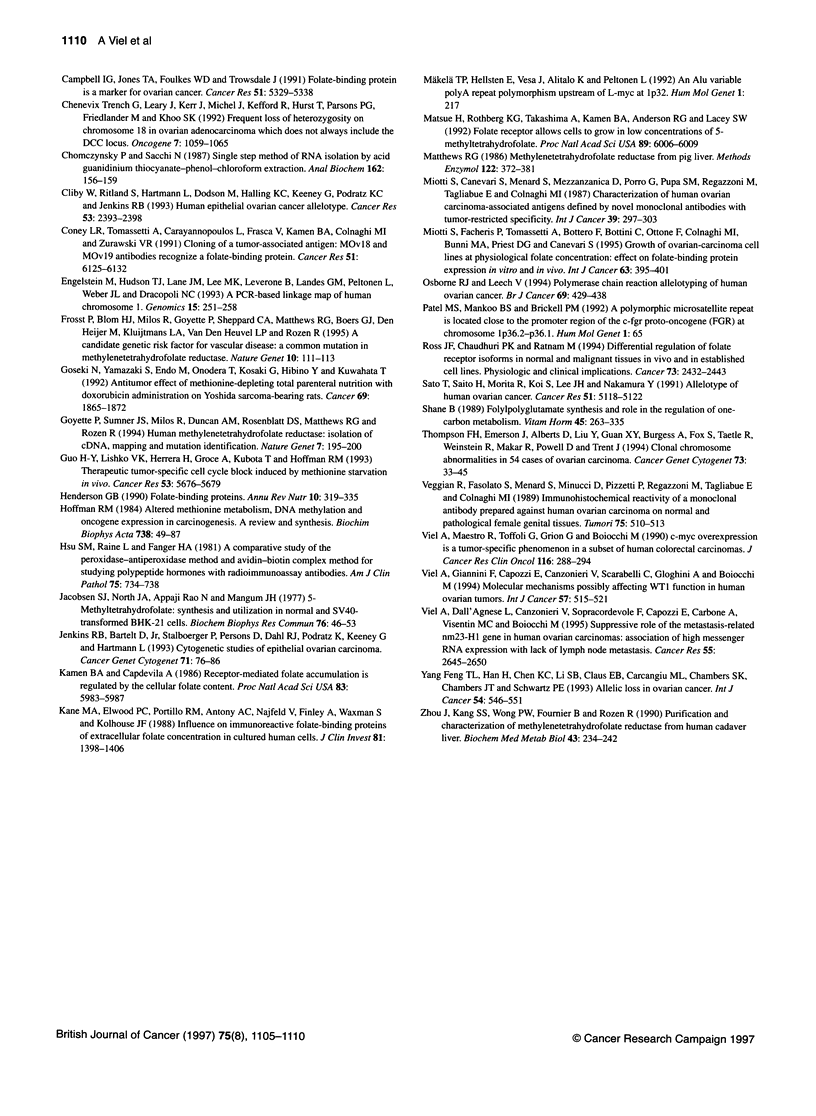# Loss of heterozygosity at the 5,10-methylenetetrahydrofolate reductase locus in human ovarian carcinomas.

**DOI:** 10.1038/bjc.1997.191

**Published:** 1997

**Authors:** A. Viel, L. Dall'Agnese, F. Simone, V. Canzonieri, E. Capozzi, M. C. Visentin, R. Valle, M. Boiocchi

**Affiliations:** Division of Experimental Oncology, Centro di Riferimento Oncologico, Aviano, Italy.

## Abstract

**Images:**


British Journal of Cancer (1997) 75(8), 1105–1110
© 1997 Cancer Research Campaign

### Loss of heterozygosity at the 5,10-methylenetetrahydrofolate reductase locus in human ovarian carcinomas

**A Viel[1], L Dall'Agnese[1], F Simone[1], V Canzonieri[2], E Capozzi[1], MC Visentin[3], R Valle[1] and M Boiocchi[1]**

Divisions of [1]Experimental Oncology 1, [2]Pathology, and [3]Department of Gynaecological Oncology, Centro di Riferimento Oncologico, 33081 Aviano, Italy

**Summary** The high-affinity folate-binding protein (FBP) is primarily involved in the uptake of the 5-methyltetrahydrofolate, and its expression may be physiologically regulated by the intracellular folate content. The overexpression of FBP on the cell surface of ovarian carcinoma cells may be responsible for an increased folate uptake. We tested the hypothesis of the existence of a defect in the 5, 10-methylenetetrahydrofolate reductase (MTHFR) in ovarian tumours that could cause reduced intracellular regeneration of the 5-methyltetrahydrofolate and induce increased FBP expression. No sequence mutations were found in the MTHFR gene, but allelic deletions of this gene were frequently detected in ovarian tumours (59%). Chromosomal losses appeared to be confined to the 1p36.3 region to which the MTHFR gene maps. Although it cannot be stated that MTHFR is the target gene of the chromosomal loss involving the 1p36.3 region, a correlation between loss of heterozygosity at this locus and decrease in MTHFR activity was shown, suggesting a role of these allelic deletions in generating a biochemical defect in folate metabolism. Further studies are needed to assess further the relationship between MTHFR and FBP overexpression, but the demonstration of the alteration of a key metabolic enzyme of the folate cycle in a subset of human ovarian tumours is in accordance with the hypothesis of an altered folate metabolism in these neoplasias and might be exploited for therapeutic purposes.

**Keywords:** 5,10-methylenetetrahydrofolate reductase; folate; folate-binding protein; loss of heterozygosity; ovarian carcinoma

Folates are a class of pteridine compounds essential for normal growth and maturation, as they are involved in one carbon atom transfer reactions, such as those necessary for the biosynthesis of methionine, deoxythymidylic acid and purines (Shane, 1989). Taking part in these metabolic pathways, folate co-enzymes are interconverted and, therefore, cellular folate uptake is only required to replace natural coenzyme degradation and to re-establish the physiological pool when cells undergo mitosis.

High-affinity folate-binding proteins (FBPs) are glycosylphosphatidylinositol (GPI)-linked membrane proteins relevant to cellular folate uptake (Henderson, 1990; Antony, 1992). Two isoforms, α- and β-FBP, were originally identified in human epidermoid carcinoma cells and normal placenta. β-FBP is generally expressed in low to moderate amounts in normal tissues. α-FBP, which is recognized by the monoclonal antibody MOv18, is moderately expressed in some normal epithelial cells but is present at higher levels in very specialized organs, such as normal fallopian tubes, adult renal proximal and distal tubules and lactating glands. Moreover, α-FBP is overexpressed in a variety of neoplastic tissues, particularly in non-mucinous ovarian carcinomas (80%) and adenomas (approximately 100%), whereas normal ovary, uterus and vagina are negative (Miotti et al, 1987; Veggian et al, 1989; Campbell et al, 1991; Coney et al, 1991; Ross et al, 1994). Such a phenotypic characteristic, although not limited to ovarian carcinomas, may suggest that these neoplastic cells have an increased metabolic requirement for folates, the uptake of which can be highly intensified by an increased expression of the high-affinity FBP receptor molecules.

Received 4 June 1996
Revised 7 October 1996
Accepted 22 October 1996

Correspondence to: M Boiocchi, Experimental Oncology 1, Centro di Riferimento Oncologico, via Pedemontana Occidentale 12, 33081 Aviano (PN) Italy

The mechanism by which FBP expression is elevated in ovarian carcinomas does not appear, in general, to involve gene amplification, but it may possibly be ascribed to the transcriptional regulation of the gene (Campbell et al, 1991). It is well known that in several cases FBP expression is physiologically regulated by the intracellular folate content (Kamen and Capdevila, 1986; Kane et al, 1988; Matsue et al, 1992; Miotti et al, 1995), most probably by the 5-methyltetrahydrofolate (5-$CH_3$-$H_4$ folate) content, since this coenzyme is the natural ligand of FBP and represents the predominant circulatory form (>90% of the overall seric folate, under physiological conditions) (Antony, 1992). Intracellular 5-$CH_3$-$H_4$ folate shortage may be consequent on limited bioavailability of extracellular folates or on biochemical defects in the intracellular regeneration or cellular retention of this folate form.

Since there was no evidence of low folate seric concentrations in patients with ovarian carcinomas (manuscript in preparation), we wondered which special condition exists in these tumours that selectively increases the demand for folates, as suggested by the overexpression of FBP. We focused our attention on the 5, 10-methylenetetrahydrofolate reductase (MTHFR), the enzyme that catalyses the intracellular regeneration of the 5-$CH_3$-$H_4$ folate (Matthews, 1986). Lack or reduced activity of MTHFR, in fact, will cause shortage of the intracellular content of the 5-$CH_3$-$H_4$ folate and possibly induce the physiological up-regulation of the FBP expression.

Table 1 Primers for MTHFR SSCP analysis

| Position[a] | Sequence[b] | bp[c] | Annealing temperature (°C) |
|---|---|---|---|
| –4 | CGGAGCCATGGTGAACGAAGCC | 211 | 60 |
| 207 | TCGAGGAGGGAAGAATTCCAGGG | | |
| 161 | TGGAATCTGGTGACAAGTGGTT | 205 | 57 |
| 365 | CCACAGTAGTTCACGGCGGT | | |
| 299 | GTGACCCTGGCTCAGACAAG | 267 | 57 |
| 565 | CACCAAACTCACTTCGGATG | | |
| 510 | GGGAGGCTTCAACTACGCAG | 292 | 57 |
| 801 | CCGAAGGGAGTGGTAGCC | | |
| 746 | TCACTTGCCCCATCGTCC | 291 | 59 |
| 1036 | GCCTGGGGTCCTCGGTC | | |
| 953 | AGAGGTGCTGAAGCGCCTGGGG | 260 | 62 |
| 1252 | GCTCCTCCTTGGGGGACTTGCT | | |
| 1187 | CTGCCTTTGGGGAGCTGAA | 285 | 58 |
| 1471 | CGTTGATGTTGGGCTGTGAG | | |
| 1431 | CCAGGGCATCCTCACCATCA | 304 | 67 |
| 1734 | GGGATCCACTACGGTGGGCT | | |
| 1693 | TTCCCTGGGCGAGAGATCAT | 293 | 67 |
| 1985 | AGGACGCAGGGTCATGGAG | | |
| 1940 | CCACCCAGAATGCGAGAGAA | 245 | 65 |
| 2184 | AGCAGAGAGTACTAGGTTCCCA | | |

[a]Positions, corresponding to 5′ end of primer, refer to MTHFR cDNA sequence no. UO9806. [b]For each primer pair, the first oligonucleotide represents the forward primer, and the second corresponds to the reverse primer. [c]Length of the PCR products.

### MATERIALS AND METHODS

### Patients and tissue samples

This study considered 89 patients with non-mucinous ovarian carcinoma who underwent surgery at the Department of Gynaecological Oncology at our centre. Tumour samples were obtained immediately after surgery and were selected at the Division of Pathology by a pathologist such that representative tissue sampling was routinely associated with sampling of adjacent tissue blocks for microscopic control examination. Peripheral blood lymphocytes or normal abdominal tissues of each individual were used as a source of normal DNA.

Five human ovarian tumour cell lines (SK-OV-3, SW 626, Caov-3, Caov-4 and NIH:OVCAR-3), obtained from the American Type Culture Collection (Rockville, MD, USA), were also introduced in this study.

### FBP expression

To determine the pattern of tumoral expression of FBP, 24 randomly chosen non-mucinous ovarian carcinomas were tested with the monoclonal antibody (MAb) MOv18 (kindly supplied by Dr S Miotti, Istituto Nazionale per lo Studio e la Cura dei Tumori, Milan, Italy). Cryostat sections were used for immunophenotyping with MOv18, by the avidin–biotin peroxidase complex (ABC) method, as described previously (Hsu et al, 1981).

### Reverse transcriptase polymerase chain reaction (RT–PCR) and single-strand conformation polymorphism (SSCP) procedures

Total RNA was extracted by the guanidinium thiocyanate–phenol–chloroform method (Chomczynsky and Sacchi, 1987) from ovarian tumour tissues and cell lines. cDNA was synthesized with the AMV reverse transcriptase (Promega, Madison, WI, USA) using random hexamers and then amplified by PCR. Primers listed in Table 1 were designed from the cDNA sequence according to numeration deposited in GenBank no. UO9806 (Frosst et al, 1995). In a first amplification round, two longer PCR products (from position –4 to 1252 and from 1187 to 2184) were obtained. An aliquot of the first PCR was then reamplified to generate ten overlapping 205–304-bp fragments spanning from position –4 to 2184. Radioactive PCR and SSCP analyses were carried out as described previously (Viel et al, 1995).

### Southern blotting

High molecular weight DNA was purified from ovarian tumours and normal tissues with an automated nucleic acid extractor (Applied Biosystems, Foster City, CA, USA). *Bgl*II-restricted DNAs were analysed as described previously (Viel et al, 1990). Blots were hybridized to a random primer $^{32}$P-labelled 1.2-kb *MTHFR* probe (portion 5′), which was obtained by RT–PCR from a normal RNA.

### Loss of heterozygosity (LOH) analysis

Restriction fragment length polymorphism (RFLP) and microsatellite analyses were performed to search for allelic deletions of sequences mapping on the short arm (p) of chromosome 1. The first experimental approach was applied to seek LOHs of the *MTHFR* gene. Genomic tumour and normal DNA (250 ng) were amplified by standard PCR (Viel et al, 1994) in the presence of 5% formamide at an annealing temperature of 51°C. The primers used were: 639, GCACTTGAAGGAGAAGGTGTC and 701, CAAAGCGGAAGAATGTGTCAG. The 83-bp PCR products were ethanol precipitated, digested with *Taq*1 and run on a 4% MetaPhor gel (FMC Bioproducts, Rockland, ME, USA). In the presence of a *Taq*1 site, two smaller fragments of 53 and 30 bp were generated. Loss of or significant reduction (at least 50%) in the intensity for one allele in the tumour DNA was interpreted as LOH. A similar analysis (RT–PCR and *Taq*1 restriction) was performed at the RNA level. In this case, tumour and normal cDNA was amplified by standard PCR (primers –4 and 1252, annealing temperature 63°C). *Taq*1 digestion produced constant bands of 95, 113 and 353 bp, and variable bands of 695 bp (allele 1) or 464 plus 231 bp (allele 2).

Microsatellite analysis was carried out by amplification of the polymorphic simple repeat regions of the *D1S160, D1S170, FGR* and L-*myc* loci, as described (Patel et al, 1992; Mäkelä et al, 1992; Engelstein et al, 1993). PCRs were performed in the presence of [α-$^{33}$P]dATP, denatured and electrophoresed on 6% denaturing polyacrylamide gels (Viel et al, 1995).

© Cancer Research Campaign 1997

**Figure 1** Representative examples of the molecular analyses that identify the Ala/Val polymorphism at position 668 and detect LOH at the MTHFR locus. (**A**) SSCP: tumours with heterozygous (1), deleted (2 and 6) and homozygous (3–5) genotypes are shown. (**B** and **C**) PCR from genomic DNA (**B**) and cDNA (**C**) followed by Taq1 restriction: allelic bands of 83 bp or 53 bp plus 30 bp (**B**), and of 695 bp or 464 bp plus 231 bp (**c**) are generated. The dimension of constant bands is indicated in parentheses. The pattern of tumour DNAs (T) is compared with that of the corresponding normal DNAs (N). Heterozygous (1), deleted (2 and 3) and homozygous (4 and 5) tumours are shown. Arrowheads indicate the allele lost in tumour DNA

**Figure 2** Representative examples of LOH analyses with the repetitive sequences associated with loci L-myc, FGR, D1S160 and D1S170. The genotype of tumour DNAs (T) is compared with that of corresponding normal DNAs (N). Heterozygous (1) and deleted (2) tumours are shown. Arrowheads indicate the allele lost in tumour DNA

### MTHFR assay

Tumour tissues were pulverized with a dismembrator (Braun, Milan, Italy). The cells were then completely disrupted by sonication in 30 mM potassium phosphate buffer (pH 7.2) and centrifuged at 13 000 *g* for 60 min. The supernatant solution containing 250 μg of protein was used for the enzyme assay, which was performed by the 5-$CH_3$-$H_4$ folate–menadione oxidoreductase method described elsewhere (Matthews, 1986), with some modifications. Briefly, the reaction mixture, in a total volume of 0.5 ml, consisted of 50 mM potassium phosphate buffer (pH 6.7), 0.3 mM EDTA, 0.33% bovine serum albumin (BSA), 10% menadione (from a saturated solution in 20% methanol) and 0.25 mM 5-[$^{14}$C]methyl-tetrahydrofolic acid (Amersham, Buckinghamshire, UK). After incubation for 1 h at 37°C, the reaction was terminated by adding 0.15 ml of a 3-mg $ml^{-1}$ dimedone solution and then boiled for 5 min. The mixtures were cooled on ice and the produced [$^{14}$C]formaldehyde–dimedone complex was extracted into 1.5 ml of toluene and dosed by scintillation counting. MTHFR activities were expressed in nmol of [$^{14}$C]formaldehyde $h^{-1}$ $mg^{-1}$ protein.

### RESULTS

### FBP expression

As expected, all the tumours tested were positive for FBP expression. A variable proportion of tumour cells ranging from 25% to 100% was stained with MOv18 (MAb), with a positivity lower than 50% in seven cases and higher than 50% in 17 other cases (data not shown).

### Mutational analysis of the MTHFR gene

Total RNA of 18 human ovarian carcinomas and five ovarian carcinoma cell lines was analysed by the RT–PCR/SSCP method. Thirteen of these 18 tumour samples were chosen from among those retaining only one allele at the *MTHFR* locus (see below). No abnormal pattern suggestive of sequence mutations was found. However, the C/T polymorphism at nucleotide 668 (but reported as nucleotide 677 in the article by Frosst et al, 1995), which converts an alanine (Ala) to a valine (Val) and creates a *Taq*1 RFLP, was detectable by this method (Figure 1). Analysis of this polymorphism at the DNA level by PCR and *Taq*1 restriction (Figure 1) showed frequencies of the allele for Val that were similar in 58 healthy blood donors (45%) and 89 ovarian carcinoma patients (47%). The frequency of the three genotypes was also superimposable in these two populations (Ala–Ala, 28% and 29%; Ala–Val, 48% and 50%; Val–Val, 23% and 22% respectively). Another common C/A polymorphism was identified at nucleotide 1289. It converts an Ala to a glutamic acid, creating an *Mbo*II site (data not shown). Finally, two sequence variations were identified at

© Cancer Research Campaign 1997

**Figure 3** Summary of allelic losses in 59 ovarian tumours. The top row lists the loci studied and their chromosomal location, but the reciprocal order of the three polymorphic sequences mapping on band 1p36.3 is not known. On the left, the numbers of tumours displaying each pattern are indicated. ▩, Heterozygous; ■, LOH; □, non-informative or not tested positions 120 and 1059 (C instead of T), which probably represent two silent polymorphisms distinguishing the Italian from the American population. In fact, we found the wild-type sequence only in an ovarian cancer patient who came from the USA.

*Bgl*II-DNA digestion and Southern hybridization to a 1.2-kb *MTHFR* cDNA probe did not show anomalous restriction fragments in the five ovarian tumour cell lines and the ten tumour samples analysed (data not shown).

### LOH analysis

The *Taq*1 RFLP of the *MTHFR* gene (mapping on chromosome 1p36.3) was used to search for allelic deletions in 89 ovarian carcinomas. The frequency of heterozygous informative patients was 49% (44/89). The analysis showed LOH at the *MTHFR* locus in a total of 26 tumours (59% of the informative cases). Of these, 11 displayed partial or total loss of the allele for Val and were defined as 'LOH Ala' because they retained the Ala allele; the other 15 deleted tumours, which lost the allele for Ala partially or totally and retained the allele for Val, were defined as 'LOH Val' (Figure 1). A similar analysis at the RNA level (RT–PCR and *Taq*1 restriction) confirmed these data in the 25 informative tumours tested (Figure 1). Seven tumours of the LOH Ala group and six tumours of the LOH Val group were also submitted to mutational screening by the RT–PCR/SSCP method.

**Table 2** Relationship between MTHFR activity and MTHFR genotype

| Tumour genotype (n)[a] | MTHFR activity (mean ± s.d.)[b] | MTHFR activity (%)[c] | Statistics[d] |
|---|---|---|---|
| Ala–Ala (14) | 16.50 ± 9.20 | 100 | |
| Val–Val (12) | 7.19 ± 3.90 | 44 | P=0.001 |
| Ala–Val (9) | 11.63 ± 4.76 | 70 | |
| LOH Val (14) | 5.82 ± 3.63 | 35 | P=0.011 |
| Ala–Val (9) | 11.63 ± 4.76 | 70 | |
| LOH Ala (5) | 10.06 ± 3.16 | 61 | NS |

[a]Number of tumours tested. [b]Expressed in nmol of [$^{14}$C]formaldehyde $h^{-1}$ $mg^{-1}$ protein. [c]Percentages are calculated with respect to the activity of Ala–Ala tumours. [d]Statistical significance was assessed by the Mann–Whitney U-test. NS, not significant.

In 59 tumours, LOH analysis was extended to other polymorphic sequences mapping on the short arm of chromosome 1 (Figure 2). Fifty-seven samples proved to be informative at one or more loci; altogether, 54% of them exhibited LOH in at least one locus on 1p (Figure 3).

The CA repeats associated with the *D1S160* and *D1S170* loci on 1p36.3 were amplified by PCR according to Engelstein et al (1993), and the observed LOH frequencies were similar to those of the *MTHFR* gene (52% and 50% respectively). A good concordance in the allelic losses of these polymorphic regions mapping on 1p was noted; in fact, *D1S160* and *D1S170* LOHs were not detected in *MTHFR* heterozygous tumours and only one *MTHFR*-deleted tumour retained both *D1S170* alleles. The polymorphism associated with the *FGR* gene on 1p36.1–2 (Patel et al, 1992) showed that heterozygosity was maintained in four cases displaying allelic losses at the above-mentioned loci. The lowest percentage of LOH was detected by the analysis of the AAAG repeat polymorphism upstream L-*myc* (1p32) (Mäkelä et al, 1992) on 46 informative cases (9/46; 20%). All the *MTHFR* heterozygous tumours also maintained heterozygosity at the L-*myc* locus; in contrast, of the cases with *MTHFR* LOH, eight retained both alleles of L-*myc* and only four showed LOH at this locus. This pattern suggested that in the majority of the cases the chromosomal loss was telomeric.

### MTHFR activity in ovarian carcinomas

The MTHFR activity was assayed in the crude extracts from 54 human ovarian carcinomas. Each test was carried out in triplicate and some tumours were tested twice, with reproducible results.

The mean activity value was 10.25 ± 7.02 nmol of [$^{14}$C]formaldehyde $h^{-1}$ $mg^{-1}$ protein. No activity was found in two cases (7%). Good quality RNAs were successfully extracted from these tumours (kept at –80°C) as a guarantee of their good preservation. The other tumours showed a variable enzyme activity, ranging from 0.58–42.38 nmol of [$^{14}$C]formaldehyde $h^{-1}$ $mg^{-1}$ protein.

The relationship between MTHFR activity and *MTHFR* genotype was studied (Table 2). The Ala–Ala homozygous tumours had the highest activity, with a mean value of 16.50 ± 9.20. In contrast, the Val–Val homozygous tumours displayed a low activity, with a mean of 7.19 ± 3.90. The Mann–Whitney *U*-test attributed a statistical significance to this distribution (*P*=0.001). Ala–Val heterozygous tumours exhibited an intermediate level (11.63 ± 4.76). Loss of one allele was proportionally associated with a decrease in MTHFR activity, with mean values of 10.06 ± 3.16 in the informative

© Cancer Research Campaign 1997

tumours showing loss of the Val allele (LOH Ala) and of 5.82 ± 3.63 in the cases displaying loss of the Ala allele (LOH Val). Comparison of heterozygous with deleted tumours showed that loss of the Ala allele, but not the Val allele, was associated with a significant decrease in MTHFR activity ($P$=0.011).

### DISCUSSION

MTHFR catalyses the reduction of 5,10-methylenetetrahydrofolate to 5-$CH_3$-$H_4$ folate, the predominant circulatory form of folates and carbon donor for the remethylation of homocysteine to methionine. The *MTHFR* human gene has been localized to chromosome 1p36.3 (Goyette et al, 1994) and codifies for a polypeptide of 77 kDa, which forms homodimers of about 150 kDa (Zhou et al, 1990). Two functional alleles of *MTHFR* have been identified in the human population because of a C to T substitution at nucleotide 668, which converts Ala residues into Val residues (Frosst et al, 1995). The allele frequency of the substitution was 0.38 in a series of 114 French–Canadian chromosomes (Frosst et al, 1995), 0.45 in 58 Italian normal blood donors and 0.47 in 89 Italian ovarian cancer patients (this study). The similar genotype frequencies at the *MTHFR* locus in the Italian healthy and affected populations suggest that the *MTHFR* genotype does not represent a risk factor in ovarian carcinogenesis.

Southern blot and SSCP analyses performed on a sample of ovarian carcinoma tissues and cell lines did not show any significant structural alterations of the *MTHFR* gene either in the portion codifying for the catalytic and substrate-binding sites or in that codifying for the regulatory sequences (Goyette et al, 1994; Frosst et al, 1995). However, our molecular analysis confirmed the existence of the above-mentioned Ala/Val allelic polymorphism (Frosst et al, 1995). Comparison of the genotype of tumour tissues with peripheral leucocytes of ovarian carcinoma patients identified 44 heterozygous patients, of whom 26 (59%) displayed deletion of one *MTHFR* allele at the tumour level (15 lost the Ala allele and 11 lost the Val allele). Such a LOH was confirmed by the analysis of two polymorphic sequences, *D1S160* and *D1S170*, which map near the *MTHFR* locus on the same 1p36.3 chromosome band (Engelstein et al, 1993); however, chromosomal losses did not extend, except in a few cases, to the 1p32 band, at which the L-*myc* locus maps (Mäkelä et al, 1992). On the whole, more than 50% of the ovarian carcinomas showed LOH in at least one of the three polymorphic markers mapping in 1p36.3, as a consequence of chromosomal deletions always including one allele of the *MTHFR* gene. This high incidence of LOH in lp has not been described previously in LOH studies performed on human ovarian carcinomas with other polymorphic sequences on chromosome 1p (Sato et al, 1991; Chenevix Trench et al, 1992; Yang Feng et al, 1993; Cliby et al, 1993; Osborne and Leech, 1994), but is in accordance with previous cytogenetic studies indicating a high frequency of structural abnormalities involving the region 1p36 (Jenkins et al, 1993; Thompson et al, 1994).

Our data therefore support the existence of a gene in 1p36.3 the deletion of which might be implicated in the oncogenic process of a significant proportion of ovarian carcinomas. Whether this gene is the *MTHFR* has to be verified. The *MTHFR* gene, although undergoing LOH in more than 50% of the ovarian carcinomas, does not completely lose its function, since no inactivating mutations were detected in the retained allele of 13 deleted tumours, as well as in five other *MTHFR* non-informative tumours and five ovarian carcinoma cell lines.

However, a functional effect at the level of cellular metabolism may be consequent to LOH at the *MTHFR* locus. The *MTHFR* alleles are concomitantly expressed in peripheral lymphocytes and ovarian carcinomas not affected by LOH at this locus, and reduced gene dosage is expected to cause a decrease in MTHFR enzymatic activity in tumours with LOH. In fact, although a considerable variability in MTHFR activity was seen in the ovarian carcinomas tested, with the highest activity in Ala–Ala homozygotes and the lowest activity in Val–Val homozygotes, the tumours affected by LOH at this locus displayed lower enzymatic activity levels than tumours of patients with the same genotype but not affected by LOH at 1p36.3.

It has been reported recently that there is a close relationship between the *MTHFR* genotype and the capability to remethylate homocysteine to methionine, demonstrating that a low MTHFR activity may really be responsible for reduced intracellular bioavailability of 5-$CH_3$-$H_4$ folate (Frosst et al, 1995). Moreover, in conditions of shortage of 5-$CH_3$-$H_4$ folate, the cell can physiologically up-modulate FBP expression in order to increase coenzyme uptake from the extracellular fluids (Kamen and Capdevila, 1986; Kane et al, 1988; Matsue et al, 1992; Miotti et al, 1995). Therefore, at least for the subset of ovarian carcinomas displaying LOH at the *MTHFR* locus, our working hypothesis seems to be confirmed, even if not directly proved. The reason is that the semi-quantitative determination of FBP expression, provided by the immunohistochemical technique, did not allow a statistical correlation between MOv18 positivity and *MTHFR* genotype and enzymatic activity. However, it should be pointed out that LOH at the *MTHFR* locus was not detected in three informative tumours of the mucinous histotype, which is usually MOv18 negative (data not shown).

The finding that FBP overexpression may also occur in ovarian carcinomas not displaying LOH at the *MTHFR* locus remains an open question, although other alterations in the folate cycles might be involved, possibly exerting a similar influence on folate metabolism and FBP expression. Studies on this topic are under way.

In conclusion, alteration of a gene coding for a key metabolic enzyme of the folate cycle, and more precisely the methionine cycle, has been demonstrated in a significant subset (59%) of human ovarian carcinomas. This should make possible the design of new therapies that take advantage of metabolic differences between ovarian tumour cells and normal cells. In particular, a possible relationship between MTHFR deficiency and methionine dependence has been proposed (Jacobsen et al, 1977), since MTHFR-defective cells are unable to synthesize 5-$CH_3$-$H_4$ folate at a rate that will satisfy the cellular demand for methionine. Such an alteration might represent a target for specific therapies, as suggested by previous studies on methionine-dependent tumours (Hoffman, 1984; Goseki et al, 1992; Guo et al, 1993).

### ACKNOWLEDGEMENTS

This work was supported in part by grants from the Italian Association for Cancer Research and CNR special project ACRO (95.01694.CT04). E Capozzi is a recipient of a fellowship from the Italian Association for Cancer Research.

### REFERENCES

Antony AC (1992) The biological chemistry of folate receptors. *Blood* **79**: 2807–2820

© Cancer Research Campaign 1997

Campbell IG, Jones TA, Foulkes WD and Trowsdale J (1991) Folate-binding protein is a marker for ovarian cancer. *Cancer Res* **51**: 5329–5338

Chenevix Trench G, Leary J, Kerr J, Michel J, Kefford R, Hurst T, Parsons PG, Friedlander M and Khoo SK (1992) Frequent loss of heterozygosity on chromosome 18 in ovarian adenocarcinoma which does not always include the DCC locus. *Oncogene* **7**: 1059–1065

Chomczynsky P and Sacchi N (1987) Single step method of RNA isolation by acid guanidinium thiocyanate–phenol–chloroform extraction. *Anal Biochem* **162**: 156–159

Cliby W, Ritland S, Hartmann L, Dodson M, Halling KC, Keeney G, Podratz KC and Jenkins RB (1993) Human epithelial ovarian cancer allelotype. *Cancer Res* **53**: 2393–2398

Coney LR, Tomassetti A, Carayannopoulos L, Frasca V, Kamen BA, Colnaghi MI and Zurawski VR (1991) Cloning of a tumor-associated antigen: MOv18 and MOv19 antibodies recognize a folate-binding protein. *Cancer Res* **51**: 6125–6132

Engelstein M, Hudson TJ, Lane JM, Lee MK, Leverone B, Landes GM, Peltonen L, Weber JL and Dracopoli NC (1993) A PCR-based linkage map of human chromosome 1. *Genomics* **15**: 251–258

Frosst P, Blom HJ, Milos R, Goyette P, Sheppard CA, Matthews RG, Boers GJ, Den Heijer M, Kluijtmans LA, Van Den Heuvel LP and Rozen R (1995) A candidate genetic risk factor for vascular disease: a common mutation in methylenetetrahydrofolate reductase. *Nature Genet* **10**: 111–113

Goseki N, Yamazaki S, Endo M, Onodera T, Kosaki G, Hibino Y and Kuwahata T (1992) Antitumor effect of methionine-depleting total parenteral nutrition with doxorubicin administration on Yoshida sarcoma-bearing rats. *Cancer* **69**: 1865–1872

Goyette P, Sumner JS, Milos R, Duncan AM, Rosenblatt DS, Matthews RG and Rozen R (1994) Human methylenetetrahydrofolate reductase: isolation of cDNA, mapping and mutation identification. *Nature Genet* **7**: 195–200

Guo H-Y, Lishko VK, Herrera H, Groce A, Kubota T and Hoffman RM (1993) Therapeutic tumor-specific cell cycle block induced by methionine starvation *in vivo*. *Cancer Res* **53**: 5676–5679

Henderson GB (1990) Folate-binding proteins. *Annu Rev Nutr* **10**: 319–335

Hoffman RM (1984) Altered methionine metabolism, DNA methylation and oncogene expression in carcinogenesis. A review and synthesis. *Biochim Biophys Acta* **738**: 49–87

Hsu SM, Raine L and Fanger HA (1981) A comparative study of the peroxidase–antiperoxidase method and avidin–biotin complex method for studying polypeptide hormones with radioimmunoassay antibodies. *Am J Clin Pathol* **75**: 734–738

Jacobsen SJ, North JA, Appaji Rao N and Mangum JH (1977) 5-Methyltetrahydrofolate: synthesis and utilization in normal and SV40-transformed BHK-21 cells. *Biochem Biophys Res Commun* **76**: 46–53

Jenkins RB, Bartelt D, Jr, Stalboerger P, Persons D, Dahl RJ, Podratz K, Keeney G and Hartmann L (1993) Cytogenetic studies of epithelial ovarian carcinoma. *Cancer Genet Cytogenet* **71**: 76–86

Kamen BA and Capdevila A (1986) Receptor-mediated folate accumulation is regulated by the cellular folate content. *Proc Natl Acad Sci USA* **83**: 5983–5987

Kane MA, Elwood PC, Portillo RM, Antony AC, Najfeld V, Finley A, Waxman S and Kolhouse JF (1988) Influence on immunoreactive folate-binding proteins of extracellular folate concentration in cultured human cells. *J Clin Invest* **81**: 1398–1406

Mäkelä TP, Hellsten E, Vesa J, Alitalo K and Peltonen L (1992) An Alu variable polyA repeat polymorphism upstream of L-myc at 1p32. *Hum Mol Genet* **1**: 217

Matsue H, Rothberg KG, Takashima A, Kamen BA, Anderson RG and Lacey SW (1992) Folate receptor allows cells to grow in low concentrations of 5-methyltetrahydrofolate. *Proc Natl Acad Sci USA* **89**: 6006–6009

Matthews RG (1986) Methylenetetrahydrofolate reductase from pig liver. *Methods Enzymol* **122**: 372–381

Miotti S, Canevari S, Menard S, Mezzanzanica D, Porro G, Pupa SM, Regazzoni M, Tagliabue E and Colnaghi MI (1987) Characterization of human ovarian carcinoma-associated antigens defined by novel monoclonal antibodies with tumor-restricted specificity. *Int J Cancer* **39**: 297–303

Miotti S, Facheris P, Tomassetti A, Bottero F, Bottini C, Ottone F, Colnaghi MI, Bunni MA, Priest DG and Canevari S (1995) Growth of ovarian-carcinoma cell lines at physiological folate concentration: effect on folate-binding protein expression *in vitro* and *in vivo*. *Int J Cancer* **63**: 395–401

Osborne RJ and Leech V (1994) Polymerase chain reaction allelotyping of human ovarian cancer. *Br J Cancer* **69**: 429–438

Patel MS, Mankoo BS and Brickell PM (1992) A polymorphic microsatellite repeat is located close to the promoter region of the c-fgr proto-oncogene (FGR) at chromosome 1p36.2–p36.1. *Hum Mol Genet* **1**: 65

Ross JF, Chaudhuri PK and Ratnam M (1994) Differential regulation of folate receptor isoforms in normal and malignant tissues in vivo and in established cell lines. Physiologic and clinical implications. *Cancer* **73**: 2432–2443

Sato T, Saito H, Morita R, Koi S, Lee JH and Nakamura Y (1991) Allelotype of human ovarian cancer. *Cancer Res* **51**: 5118–5122

Shane B (1989) Folylpolyglutamate synthesis and role in the regulation of one-carbon metabolism. *Vitam Horm* **45**: 263–335

Thompson FH, Emerson J, Alberts D, Liu Y, Guan XY, Burgess A, Fox S, Taetle R, Weinstein R, Makar R, Powell D and Trent J (1994) Clonal chromosome abnormalities in 54 cases of ovarian carcinoma. *Cancer Genet Cytogenet* **73**: 33–45

Veggian R, Fasolato S, Menard S, Minucci D, Pizzetti P, Regazzoni M, Tagliabue E and Colnaghi MI (1989) Immunohistochemical reactivity of a monoclonal antibody prepared against human ovarian carcinoma on normal and pathological female genital tissues. *Tumori* **75**: 510–513

Viel A, Maestro R, Toffoli G, Grion G and Boiocchi M (1990) c-myc overexpression is a tumor-specific phenomenon in a subset of human colorectal carcinomas. *J Cancer Res Clin Oncol* **116**: 288–294

Viel A, Giannini F, Capozzi E, Canzonieri V, Scarabelli C, Gloghini A and Boiocchi M (1994) Molecular mechanisms possibly affecting WT1 function in human ovarian tumors. *Int J Cancer* **57**: 515–521

Viel A, Dall'Agnese L, Canzonieri V, Sopracordevole F, Capozzi E, Carbone A, Visentin MC and Boiocchi M (1995) Suppressive role of the metastasis-related nm23-H1 gene in human ovarian carcinomas: association of high messenger RNA expression with lack of lymph node metastasis. *Cancer Res* **55**: 2645–2650

Yang Feng TL, Han H, Chen KC, Li SB, Claus EB, Carcangiu ML, Chambers SK, Chambers JT and Schwartz PE (1993) Allelic loss in ovarian cancer. *Int J Cancer* **54**: 546–551

Zhou J, Kang SS, Wong PW, Fournier B and Rozen R (1990) Purification and characterization of methylenetetrahydrofolate reductase from human cadaver liver. *Biochem Med Metab Biol* **43**: 234–242

© Cancer Research Campaign 1997